# Prediction of Optimum Process Parameters Fabricated by Direct Laser Interference Patterning Based on Central Composite Design

**DOI:** 10.3390/ma13184101

**Published:** 2020-09-15

**Authors:** Mikhael El-Khoury, Bogdan Voisiat, Tim Kunze, Andrés Fabián Lasagni

**Affiliations:** 1 Institut für Fertigungstechnik, Technische Universität Dresden, George-Bähr-Str. 3c, 01069 Dresden, Germany; bogdan.voisiat@tu-dresden.de (B.V.); andres_fabian.lasagni@tu-dresden.de (A.F.L.); 2 Fraunhofer-Institut für Werkstoff- und Strahltechnik IWS, Winterbergstr. 28, 01277 Dresden, Germany; tim.kunze@iws.fraunhofer.de

**Keywords:** nanosecond laser, direct laser interference patterning, design of experiments, central composite design, morphological filtering, surface texture homogeneity, micro structuring, bearing steel

## Abstract

In this study, we report on the optimization of the direct laser interference patterning process by applying the design of experiments approach. The periodic line-like microstructures of a 8.50 µm spatial period were fabricated by a two-beam interference setup with nanosecond laser pulses, varying laser fluence, pulse overlap, and hatch distance. Central composite design with three factors and five levels was implemented to optimize the required number of experiments. The experimental and numerical results show the impact of various structuring process parameters on surface uniformity. The responses measured are the structure height, height error, and waviness of the pattern. An analysis of the microstructures on the patterned surface was conducted by confocal microscopy and scanning electron microscopy. A 3D-characterization method based on morphological filtering, which allows a holistic view of the surface properties, was applied, and a new qualification scheme for surface microstructures was introduced. Empirical models were also developed and validated for establishing relationships between process parameters and performance criteria. Multi-objective optimization was performed to achieve a minimal value of structure height errors and waviness.

## 1. Introduction

The functionalization of technical surfaces by producing deterministic topographies today represents an innovation carrier of modern materials engineering. Nature has shown to be the best surface engineer, and the design of these textured surfaces often follows a biomimetic approach motivated by natural designs [1,2]. Therefore, mimicking natural designs helps in understanding the role of surface microstructures and to correlate topographies to macroscopic surface properties. For instance, these well-defined and highly-oriented structures fabricated at the micron and sub-micron scale on surfaces of modern industrial products enable a clear innovation potential to improve products performance significantly [3]. This upgrade concerns a wide range of applications, such as antifouling [4], wetting control, [5,6], tribology [7,8,9,10], electrical conductivity improvement [11], cell adhesion [12], as well as surface optical appearance alteration [13,14]. However, the reproduction of these versatile surface structures represents one of the most significant technical challenges today due to their complexity.

In this frame, non-contact manufacturing processes, such as laser-based microprocessing, arose as an extremely viable approach for mimicking natural surfaces because it cannot only provide both the required technological and economic aspects but also ensures the capability to produce high-resolution features [15]. Nowadays, the most prominent laser patterning approaches are direct laser writing, laser-induced periodic surface structures, and direct laser interference patterning [15].

**Figure 1:**
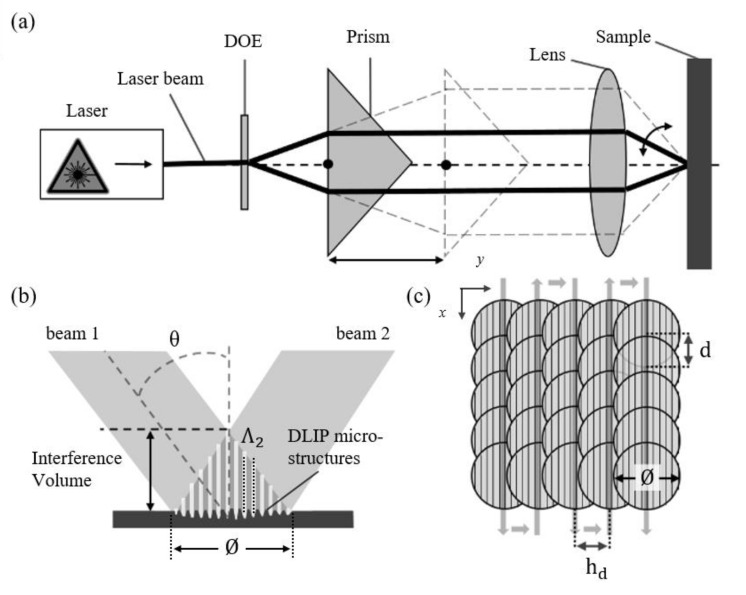
(**a**) Schematic representation of DLIP optical setup; (**b**) schematic view of the interaction zone (with a diameter *Ø)* of two beams intercepting at angle 2θ and forming an interference pattern with a particular period of $\Lambda_{2}$; (**c**) distribution of pulses during the DLIP process: Ø, d, and *h_d_* denote the laser beam diameter (at level 1/e^2^), pulse overlap, and hatch distance, respectively.

In direct laser writing, the focused laser beam is scanned over the material surface employing pulse-to-pulse strategies, and the resolution is limited by optical diffraction at the focal position. In the case of laser-induced periodic surface structures, repetitive patterns are obtained based on self-organization processes with feature sizes even below the diffraction limit and in the range of the wavelength, or even much smaller [16]. On the other hand, direct laser interference patterning (DLIP) takes advantage of the physical principle of interference of coherent light waves to produce periodic structures on a surface by transferring the pattern shape directly to the material when sufficient laser energy per unit of area (fluence) is applied [17,18,19,20]. The interference patterns are formed by splitting a coherent laser beam into multiple beams and hereafter overlapping them on the samples’ surface (see Figure 1a,b). Therefore, due to constructive and destructive interference, a specific intensity pattern is obtained, and the size of the periodic structures can be controlled by varying the angle between the interfering beams and the laser wavelength, according to Equation (1):

(1) $$\Lambda_{2}=\frac{\lambda }{2\sin\left(\theta \right)}$$

Despite the simple optical arrangement required for DLIP for fabricating periodic structures, it is also important to assure in several cases a uniform distribution of these structures to guarantee, overall, a specific surface functionality.

As reported in several studies, the quality of the final surface morphology strongly depends on laser-material interaction and, thus, on the used laser beam profile and the structuring strategy [21,22]. During the DLIP process, laser-material interaction occurs predominantly at the positions corresponding to the interference maxima, inducing various metallurgical processes, such as melting, ablation, and recrystallization [20]. During nanosecond-pulsed laser processing of metals, structuring mechanisms are mainly based on recoil vapor pressure as well as on Marangoni convection, which have an effect on the overall picture of melt flow [22,23,24]. In most of the commercial laser systems, the laser beam profile has a Gaussian distribution (${\mathrm{TEM}}_{00}$ profile), which necessitates the use of advanced processing strategies, such as consecutively overlapped irradiation of interfering laser pulses in order to form homogeneous structures on large surface areas. For example, Aguilera et al. [22] used this technology and varied different processing parameters in order to produce homogeneous structures on a large surface area. Experiments were carried out in such a way that one process parameter (factor) was applied and varied through different values (levels), then the response was analyzed, whereas the other factors remain unaffected. This procedure is called one variable at a time [25]. For instance, in the mentioned study, discrete values of overlap distance, hatch distance, and fluence were chosen for specific special periods, and it was found that for fluence F = 1.42 J/cm^2^, 98.5% overlap and hatch distance of 20 µm, for 120 µm beam diameter spot, the structured surface was uniform and, thus, obtaining a homogeneous periodic pattern [22]. However, this approach is time-consuming since several variables have to be to screened independently, resulting in a large number of experiments. Furthermore, the number of experiments increases exponentially with the increase of the number of the factors and their levels.

Additionally, in one variable at a time method, it is impossible to single out the effects of factors interaction that can only be observed when varying multiple factors at the same time. Furthermore, by applying one variable at a time approach, it is possible to miss a process window with optimal settings of factors that will give the desired response. Therefore, these factors should not be examined independently. They should be taken into account simultaneously and must be investigated together since one factor might depend on the level of the other factor. In this case, a more effective approach is a statistical design of the experiment, which aims to decrease the number of experiments and to study the effect of interactions between different factors. For this purpose, many experimental design methods, namely, Plackett and Burman, factorial, Box–Behnken, and central composite design (CCD), have been developed [26,27,28]. Among these experimental methods, CCD developed by Box and Wilson is a very efficient experimental design method to reduce the number of experiments in the studies with a large number of factors and levels [28]. CCD has more advantages compared to other experimental design methods. For instance, it provides high-quality predictions in studying linear and quadratic interaction effects of factors influencing a system. Whereas interactions, unobserved in Plackett and Burman’s experimental methods and Box–Behnken has less coverage than in the case of CCD [28,29]. Therefore, CCD has been widely used in the fields of engineering and science [29,30,31,32,33,34]. The CCD consists of three main parts and of 2*^k^* + 2*k* + *m* runs.

**Figure 2:**
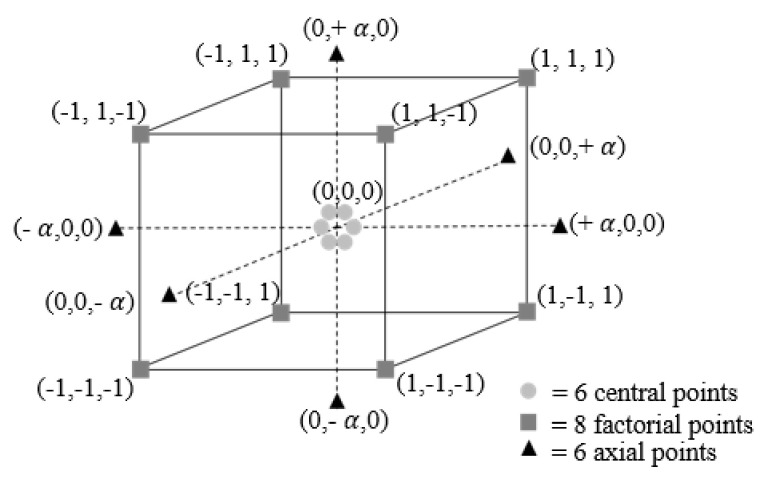
Central composite design with three factors and five levels each.

The factorial part of CCD is a two-level design with *2^k^* factorial points at the corners of a cube denoting its design in space as shown in Figure 2. For a cube design, the number 2 in the last expression results from the amount of levels, and *k* is set to 3, representing the number of factors. The other part of CCD is fixed at the center of the design space and consists of *m* center points (see Figure 2), which represent the middle levels of all the factors investigated. The replication of these points allows estimation of experimental error, detection of curvature in the fitted data, and checking the adequacy of the model. Consequently, the replication of the entire experimental design is not required [28].

The last part comprising CCD is to define the axial points. There are *2k* axial points in a CCD, and they establish new extreme levels (the lowest and the highest level) for each factor. The distance between the axial and center points is denoted by ± α value, where $\alpha =2^{k/4}≅1,68179$ for *k* = 3. This value gives rotatability to the design, which ensures that the variance of the model prediction is constant at all points equidistant from the design center [35]. It makes the CCD method able to explore the wide process space and to capture a strong curvature for studying the effects of the interactions between the design factors on the model [36,37,38].

In this contribution, we present the optimization and fabrication of homogeneous periodic surface microstructures on bearing steel (100Cr6) using the design of experiments approach and employing a two-beam-DLIP setup with an infrared (IR) nanosecond laser. Since it has been shown that the improvement of the structure homogeneity is more dependent on the strategy used during the experiments than on the pulse duration of the laser source, the aim is to optimize the DLIP process parameters such as laser peak-fluence, pulse overlap, and hatch distance (see Figure 1c) with respect to the structures’ height and surface texture homogeneity by performing the CCD method. The surface topography is characterized using confocal microscopy and scanning electron microcopy analysis. Furthermore, a 3D-characterization method for measuring the pattern homogeneity was applied based on morphological filtering [39,40,41,42], which allows a holistic view of the surface properties, and a new qualification approach of DLIP surface structures was introduced. The method presented here is of significant relevance to assure, in the future, a certain performance over the whole treated area as well as to permit in relevant industrial processes to quantitatively describe the produced topography in terms of homogeneity. It is required, for instance, for quality management.

## 2. Materials and Methods

### 2.1. Materials

The laser texturing experiments were performed on a hardened bearing steel surface (100Cr6), commonly used for the manufacturing of automotive components. The test samples were cut into round shaped substrates with a diameter of 40 mm and 10 mm thickness. Each test surface was ground, resulting in surface roughness S_a_ of 0.74 µm (DIN-ISO 25178). Before the laser treatment, all samples were cleaned using isopropanol.

### 2.2. Nanosecond Two-Beam-DLIP Setup and Process Strategy

The laser experiments were carried out using a two-beam DLIP-μFab system (developed by Fraunhofer IWS, Dresden, Germany), which produces the interference spot that contains a line-like intensity pattern. The system is equipped with a pulsed Q-switched Nd:YLF laser (Laser-export Tech- 1053 Basic, Moscow, Russia), operating at 1053 nm wavelength and providing 12 ns pulses with pulse energy up to 290 μJ at 1 kHz. The laser emits the fundamental transverse mode (${\mathrm{TEM}}_{00}$) with a laser beam quality factor of M^2^ < 1.2. This system also includes a compact DLIP optical head, where the main beam is split into two beams using a diffractive optical element (DOE), then the beams are parallelized by a prism and finally overlapped using a lens with a focal distance of 40 mm (see Figure 1a). Such an optical configuration provides the fully-automatic control of the spatial period of the interference profile between 1.45 μm and 8.50 μm by varying the incidence angle of the beams $\left(\theta \right)\;$ on the sample, which, in turn, is realized by moving the prism with respect to the DOE [43].

In this study, different pulse peak-fluences $F_{p}$ (ranging from 4.97 J/cm^2^ up to 7.07 J/cm^2^) were used to determine the energy range needed for uniform structuring of bearing steel surface. The peak-fluence on the interference spot was calculated according to Equation (2):

(2) $$F_{p}=\frac{2n\cdot P}{f\cdot \pi \cdot w^{2}\;},$$

where n is a number of interfering beams, P and f is power and repetition rate of the laser, respectively, and w denotes a beam radius (at level 1/e^2^). In this work, the beam radius *w* was determined using the D-squared method and was equal to 52.5 μm [44,45].

To structure larger areas, the sample was moved by XY-stages (PRO Series, Aerotech Ltd., Tadley, UK) with linear speeds ranging from 0.22 cm/s to 1.60 cm/s. The arrangement of the pulse position in the structuring process is illustrated schematically in Figure 1c. This process occurs consecutively such that first overlap in the x-direction (feed direction) occurs then substrate is moved in the y-direction by hatch distance, making an overlap also in y-direction. Notable that x-direction was chosen as feed direction since it is parallel to the orientation of the line-like pattern, and thus guarantees a well-defined periodic structure. The pulse-to-pulse overlap in x-direction is denoted as *P_O_* (%) and represented as a function of pulse-to-pulse separation distance *d* (*d =* v_scan_·*f*, where v_scan_ is the scanning speed of *x*-axis and *f* is the used laser pulse repetition rate) and the laser beam diameter Ø using Equation (3):

(3) $$P_{O}\;\left[\%\right]=\left(1-\frac{d}{Ø}\;\right)\cdot 100\%$$

In the experiments, the pulse-to-pulse overlap was varied between 82.56% and 98.52%. On the other hand, the hatch distance *h_d_* (distance between the vertical lines) was controlled by shifting the sample with the *y*-axis in such a way that *h_d_* was always kept multiple of the spatial period. In addition, hatch distance *h_d_* was varied from 25.5 µm up to 68 µm. The laser pulses overlap in y-direction is also introduced, and it can be represented as a function of hatch distance *h_d_* and the laser beam diameter Ø (at level 1/e^2^) using Equation (4):

(4) $$H_{D}\;\left[\%\right]=\left(1-\frac{h_{d}}{Ø}\;\right)\cdot 100\%$$

### 2.3. Central Composite Design Method

In the present study, the experimental plan with the variation of parameters and statistical analyses of the experimental data was carried out using MINITAB 18 statistical package. The experiments were designed based on the CCD method. In Figure 2, each axis corresponds to a factor while each point on the cube represents certain levels. The three selected factors are fluence (*X*_1_), pulse overlap (*X*_2_), and hatch distance overlap (*X*_3_). It is recommended that six center points are taken in a CCD with three factors [35].

As was stated before, the center point is replicated to find the experimental error, and so the replication of the entire experimental design is usually not required. However, during pretests model variability (i.e., how well the regression model fits the experimental data) determined by the *R*^2^ factor was <80%, which is less than the acceptable value according to [38]. Therefore, it was decided to make a replication of the entire experimental design in order to increase the statistical significance of the measurements and improve the models’ regression fit. As a result, the factorial, center and axial points in a CCD method build up an experimental design with five levels for each factor and three replicates, making 60 runs in total (3(2*^k^* + 2*k* + *m*) = 3(8 + 6 + 6) = 60). The designed 60 experiments of the DLIP process were conducted in random order to exclude any bias in the response variables and to avoid a systematic error associated with the specific factor combinations as it was suggested in [28,37].

The experimental plan with the coded and uncoded levels of design factors is presented in Table 1. The low, middle, and high levels of each factor are coded as −1, 0, and +1, respectively, while the lowest and the highest levels are coded as −1.5 and +1.5.

The mathematical relationship of Y response on the corresponding factors is expressed by the following second-order polynomial equation [36,37]:

(5) $$Y=\beta_{0}+\overset{k}{\underset{i=1}{\sum }}\beta_{i}X_{i}+\overset{k}{\underset{i=1}{\sum }}\beta_{ii}X_{i}{}^{2}+\overset{k}{\underset{i=1}{\sum }}\overset{k}{\underset{j>i}{\sum }}\beta_{ij}X_{i}X_{j}+\varepsilon \;;\;i=1,\;2,\;\ldots ,\;k;\;j=2,\;\ldots ,\;k;\;i\neq j,$$

where Y is the observed response value (structures height, structure-height-error, and waviness percentage); *X_i_* and *X_j_* are the coded values of factors, *β*_0_ is the constant, *β_i_*, *β_j_*, and *β_ij_* are the linear, quadratic, and interaction coefficients respectively, *k* is the number of the factors, and *ε* is the error term. MINITAB was also used to generate the Pareto charts, response surface, contour plots of factors, as well as the optimization plots. Excluding the control factors, each test was carried out under the same experimental conditions in the ambient environment without post-treatment.

### 2.4. Surface Characterization

The 3D surface topography of structured samples was characterized using confocal and coherence scanning interferometry microscopy (Sensofar, S Neox non-contact 3D Surface Profiler, Barcelona, Spain) employing a 50× magnification objective, with a lateral and vertical resolution of 340 nm and 4 nm, respectively. Using this objective, a total area of 351 µm × 264 µm could be recorded in each measurement. Afterward, using the software MountainsMap^®^ 7.4 (Digital Surf, Besançon, France), the surface profiles of the recorded topographies are extracted applying morphological filters (ISO16610-14), and the topographical 3D roughness parameters are calculated by the recognized measurement (ISO 25178-2). In addition, topographical measurements have been carried out using a scanning electron microscopy at an operating voltage of 15 kV (JEOL, JSM 6610LV, Tokyo, Japan).

To describe the homogeneity of the fabricated structures, two topographical parameters were used, namely, structure height error and waviness. Waviness shows how the structure height is changing at distances larger than the structure period. This kind of structure inhomogeneity is caused by too large a hatch distance between the laser pulses [22]. However, when the structure hatch distance is small enough, the waviness of the structure becomes close to zero. At this point, the homogeneity of the structure is described better by the structure height error parameter.

### 2.5. Development of a Topographical Analysis Method

**Figure 3:**
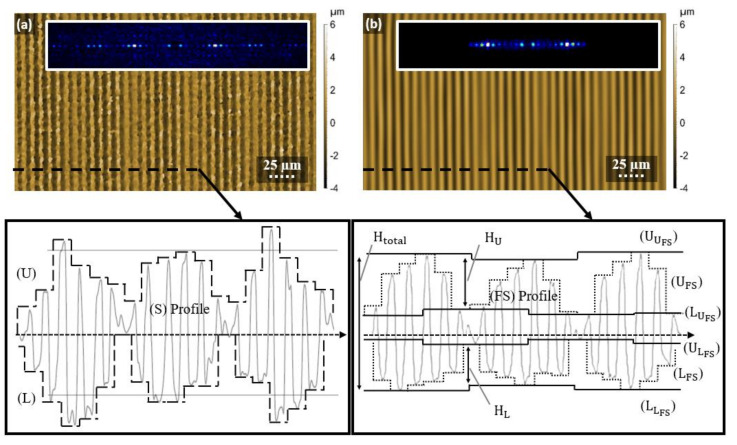
Topography images of (**a**) measured DLIP surface and (**b**) filtered surface with corresponding roughness profiles. The insets show Fourier transform of each of the surfaces.

To perform a homogeneity analysis of the structured surface, an analysis algorithm, based on the extraction of surface envelopes from the measured surface (S) using morphological filters (MF), was developed. A morphological filter is based on two morphological operations, dilation, and erosion, that work using a structuring element (SE) of a given size [41,42]. In this case, the SE was set to the size of the structure period Λ. By applying the dilation (dMF) and erosion (eMF) morphological filtering the upper (U) and lower (L) structure envelopes were achieved, respectively (profile inset Figure 3a). The U and L envelopes represent the distribution of heights and depths of structure hills and valleys, respectively. Accordingly, $H=\left|U-L\right|$ represent the fabricated structure height distribution. Then, the average structure height (h) and its error (h_err_) can be calculated by finding an average and root mean square of H, respectively. Equations (6) and (7) represents the mathematical procedures described in this paragraph:

(6) $$U=eMF_{\mathrm{SE}=\Lambda }\left(S\right);\;L=dMF_{\mathrm{SE}=\Lambda }\left(S\right);\;H=\left|U-L\right|;$$

(7) $$h_{err}=\frac{RMS\left(H\right)}{H}\cdot 100\%.$$

In order to separate structure height error from the waviness calculations, special filtering is applied to the measured surface *S*. Spatial filtering is performed by calculating the fast Fourier transform of the *S*, then applying spatial filter and finally performing the inverse Fourier transform on the filtered Fourier space data:

(8) $$\left(i\right):FS\;=\;iFFT\left(S\_Filter\;\left(FFT\left(S\right)\right)\right)$$

As it was mentioned in Section 2.4, the waviness is proportional to the hatch distance between the laser pulses. Therefore, the waviness is measured by using similar technique described in previous paragraph, but with structuring element of the MF filter equal to the hatch distance that was used to form the structure. Altogether, the calculation of the waviness consist of few steps. First, the upper ($U_{FS}$) and lower ($L_{FS}$) envelops of the filtered surface (FS) are calculated:

(9) $$\left(\mathrm{ii}\right):U_{FS}=eMF_{\mathrm{SE}=\Lambda }\left(FS\right);\;\left(\mathrm{iii}\right):L_{FS}=dMF_{\mathrm{SE}=\Lambda }\left(FS\right).$$

Then the upper and lower envelopes of $U_{FS}$ and $U_{FS}$ are calculated with $SE=h_{d}$:

(10) $$\left(\mathrm{iv}\right):U_{U_{FS}}=eMF_{\mathrm{SE}=h_{d}}\left(U_{Fs}\right);\;\left(v\right):U_{U_{FS}}=dMF_{\mathrm{SE}=h_{d}}\left(U_{Fs}\right);\;\;\left(\mathrm{vi}\right):U_{L_{FS}}=eMF_{\mathrm{SE}=h_{d}}\left(L_{Fs}\right);\;\left(\mathrm{vii}\right):\;L_{L_{FS}}=dMF_{\mathrm{SE}=h_{d}}\left(L_{Fs}\right).$$

Finally, the waviness of the function is expressed by Equation (11):

(11) $$H_{U}=\left(U_{U_{FS}}-U_{U_{FS}}\right);\;H_{L}=\left(U_{L_{FS}}-L_{L_{FS}}\right);\;H_{Wav}=H_{U}+H_{L};\;H_{total}=U_{U_{FS}}-L_{L_{FS}};\;\;W\left[\%\right]=\frac{H_{wav}}{H_{total}}100$$

An example of this procedure is shown in Figure 3, in which explanation is done on 2D profiles instead of 3D for simplicity. It shows the topography image of a DLIP structured surface measured by confocal microscope in Figure 3a,b represents its filtered Fourier space. The inserts in both images show the fast Fourier transforms of the corresponding topographies and visualizes how the smallest scale elements from surface (a) that form the noise (unreal measured peaks, solidified debris, dirt particles, etc.) are filtered by using the special filtering method (ISO25178), leaving only the surface waviness and the smaller line-like pattern. Additionally, the extracted profile pictures of selected areas are shown for both cases, showing the different topography feature indicators described in the previous section.

## 3. Results and Discussions

### 3.1. Statistical Analysis of Results

The surface of the steel sample was patterned using the two-beam DLIP configuration. The µsec processing strategy was already described in Section 2.2. Areas with a size of 3 × 3 mm were structured with different processing parameters. The varied process parameters were (i) hatch distance, (ii) pulse overlap, and (iii) peak-laser-fluence (see Table 1).

**Figure 4:**
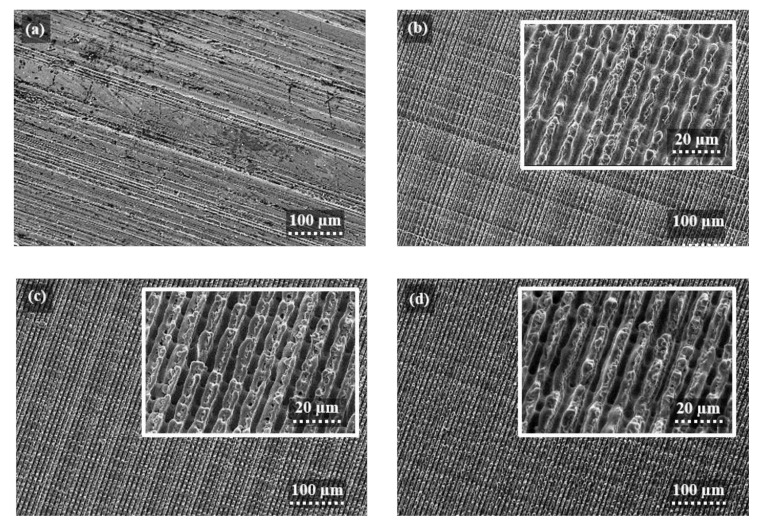
Scanning electron micrographs of initial surface topography (**a**), and the nanosecond DLIP structures produced on 100Cr6 steel using spatial period Λ = 8.50 μm, and the corresponding processing parameters: F = 6.02 J/cm^2^, P_O_ = 90.54%, H_D_ = 58% (**b**), F = 6.02 J/cm^2^, P_O_ = 98.52%, H_D_ = 58% (**c**), and F = 6.72 J/cm^2^, P_O_ = 98.52%, H_D_ = 70% (**d**). The insets show the corresponding magnification of the topographies.

The scanning electron micrographs of three exemplary patterned surfaces are shown in Figure 4b,d. The surfaces were processes with the following process parameters: F = 6.02 J/cm^2^, P_O_ = 90.54%, H_D_ = 58% (Figure 4b), F = 6.02 J/cm2, P_O_ = 98.52%, H_D_ = 58% (Figure 4c) and F = 6.72 J/cm^2^, P_O_ = 98.52%, H_D_ = 70% (Figure 4d). The reference topography with initial surface roughness is presented in Figure 4a. Due to the high roughness of the initial samples’ surface, the grinding stripes are still be visible after the laser process (see features perpendicular to the line-like structure in Figure 4b). In this case, low laser fluence and pulse overlap were used, which leads to the formation of shallow structures with height in the range of surface roughness. Differently, at higher fluence values and/or increased pulse overlap, the initial surface roughness is flattened as it can be seen from Figure 4c,d. Furthermore, the high magnification images depicted in the insets of Figure 4c,d clearly show that during nanosecond-pulsed laser treatment besides ablation phenomena, redeposition of the molten material driven by Marangoni convection and recoil vapor pressure [22,24] takes part in the structuring mechanism, creating structures with higher aspect ratios (height to spatial period ratio) [46].

In addition, to the periodic microstructures produced by the interference pattern, also waviness of the structured topography is very pronounced for the used hatch distances 42.5 µm, which corresponds to H_D_ = 58% in Figure 4b,c. This structure topography can be explained since the interference patterns are obtained using two overlapped beams with a Gaussian intensity distribution (${\mathrm{TEM}}_{00}$), which results in an interference pattern also with a Gaussian distribution [47,48]. Therefore, due to the Gaussian beam profile, the fluence in the center of the spot is higher, which lead to high cumulative energy and, thus, strong ablation and a significant quantity of molten material occur. Furthermore, since the sample is moved in the x direction (parallel to the orientation of the interference lines) and the pulses are overlapped, which leads to deeper structures at the central area as well as shallow structures at the periphery, producing the larger repetitive structure. Moreover, the repetitive length of waviness modulation is equivalent to the hatch distance used as reported in [22].

**Figure 5:**
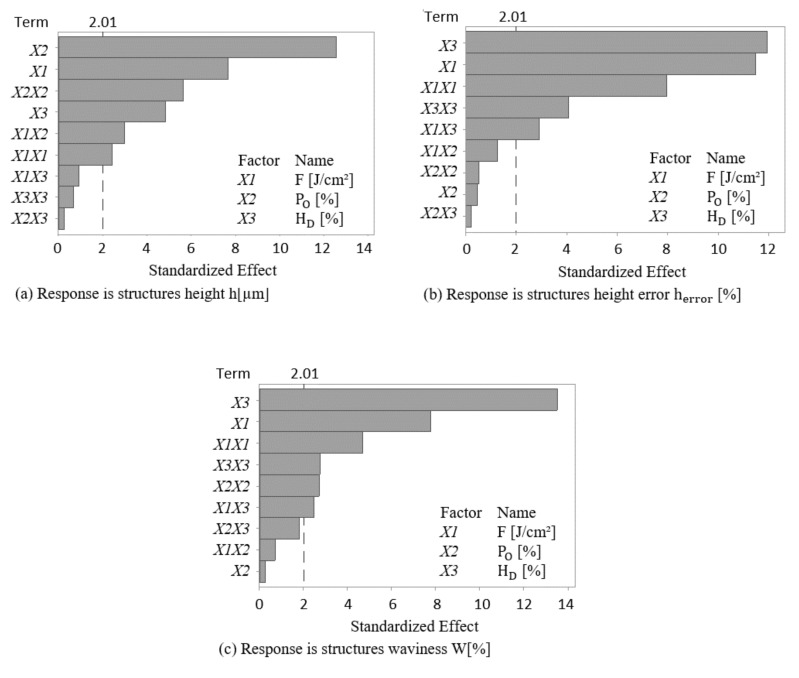
Pareto charts of the standardized effect of (**a**) structures height, (**b**) structures height error, and (**c**) surface waviness.

For determining the general surface texture homogeneity, the experimental results obtained by using the topographical analysis method described in Section 2.5 were analyzed statistically. Analysis of variance (ANOVA) was performed to identify the significance of the factors and their interactions as well as for estimating the adequacy of the model. Pareto charts of the ANOVA standardized effect estimates are presented in Figure 5. A Pareto chart is very useful for reviewing a large number of factors and for presenting the magnitude and the importance of the effects. In the plotted diagrams, a vertical reference line indicates the minimum magnitude of statistically significant effects, which depends on the significance level denoted by α and set in our model to 0.05 (5% of risk) by convention described in [49]. In addition, the bars that cross the reference line are statistically significant.

For instance, in Figure 5a where different standardized effects in the structure height response are shown, it is worth to point out that pulse overlap (*X*_2_) as well as laser-fluence (*X*_1_) have the highest effect on the formation of structure height. Other significant effects according to the developed model are square interaction of each of pulse overlap (*X*_2×2_) and laser-fluence (*X*_1_*X*_1_) as well as their two-way interaction (*X*_1_*X*_2_). Other relevant single effect which is worth to mention is the hatch distance (*X*_3_), whereas the square interaction of hatch distance (*X*_3_*X*_3_) as well as the two-way interactions between hatch distance with each of fluence (*X*_1_*X*_3_) and pulse overlap (*X*_2_*X*_3_) are not significant at the 0.05 level with the current model terms.

In the same manner, the Pareto chart of Figure 5b, shows the standardized effects for structure height error. In this case, almost all factors have significant effects at the 0.05 level on the model, except those of single, square and two-way interactions of pulse overlap (*X*_2_, *X*_2_*X*_2_, *X*_2_*X*_3_, and *X*_1_*X*_2_). Finally, in the Pareto chart shows in Figure 5c, it is possible to see that for the standardized effects for waviness, almost all factors have significant effects at the 0.05 level, except the effect of single interaction of pulse overlap (*X*_2_) as well as the effects of each of the two-way interactions between pulse overlap with each of fluence (*X*_1_*X*_2_) and hatch distance (*X*_2_*X*_3_).

Moreover, from the Pareto charts, it is possible to determine the most influencing factors among all the relevant candidates. This is visible when calculating the contribution in percentages of each factor for the different developed models, which is shown in Table 2. The results show that pulse overlap (*X*_2_), followed by fluence (*X*_1_), have the highest contribution to the structure height, whereas in the case of height error and waviness, the hatch distance (*X*_3_) followed by fluence (*X*_1_) have the highest contributions.

The regression equations obtained by removing insignificant terms from the model for each of the responses are given as follows:

(12) $$\mathrm{Model}\;\#1:\;S=0.883\;and\;R^{2}=84.82\%;\;h\;\left(m\right)=275.50+2.26X_{1}+6.41X_{2}+0.06X_{3}-0.79X_{1}X_{1}\;-0.03X_{2}X_{2}+0.15X_{1}X_{2}$$

(13) $$\mathrm{Model}\;\#2:\;S=7.740\;and\;R^{2}=87.54\%;\;h_{error}\;\left(\%\right)=1368-328.60X_{1}+0.11X_{2}-9.22X_{3}+22.90X_{1}X_{1}+0.04X_{3}X_{3}+0.55X_{1}X_{3}$$

(14) $$\mathrm{Model}\;\#3:\;S=8.954\;and\;R^{2}=84.22\%\;W\;\left(\%\right)=1943-167.40X_{1}-27.30X_{2}-1.92X_{3}+15.13X_{1}X_{1}\;+0.15X_{2}X_{2}+\;0.03X_{3}X_{3}-0.53X_{1}X_{3}$$

The positive and the negative sign in Equations (12)–(14) represent the synergistic and antagonistic effects of the factor on the response for each of the models, respectively. Furthermore, the determination coefficient (*R*^2^) of model #1 is 84.82%, showing a acceptable agreement with the experimental data. Moreover, the standard error of the regression (S) of model #1 is 0.883. This means that this statistical model can explain 84.82% variability in the response and that the average distance of the structure height data points from the fitted line is about 0.883 µm. It is worth mentioning that S is represented in the units of the dependent variable. Likewise, in model #2 the determination coefficient (*R*^2^) and the standard error of the regression (S) are equal to 87.54% (>80%) and 7.74%, respectively. Finally, for model #3 the corresponding determination coefficient and the standard error of the regression are as following *R*^2^ = 84.22% (>80%) and S = 8.954%. It can be concluded from both values that each of the models is statistically significant.

**Figure 6:**
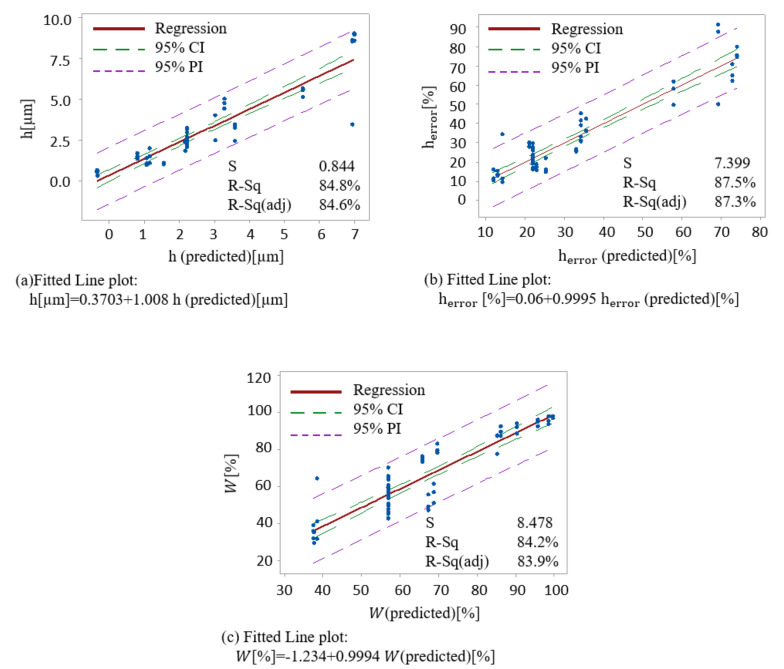
Correlation fitted line plots between experimental and predicted values for each of (**a**) structures height, (**b**) structures height error, and (**c**) surface waviness.

In order to compare the results from the models with experimental data, the correlation graphs were analyzed showing a good correlation, as shown in Figure 6. In the plots, the upper and lower limits of the confidence and prediction intervals are displayed as dashed lines. The confidence interval displays the range of 95% of possible values for the mean response, whereas the prediction interval displays the range of 95% of possible values for a single new observation. The *R*^2^ value in each of the correlation graphs, shows that the developed model can explain >80% variability in the response.

**Figure 7:**
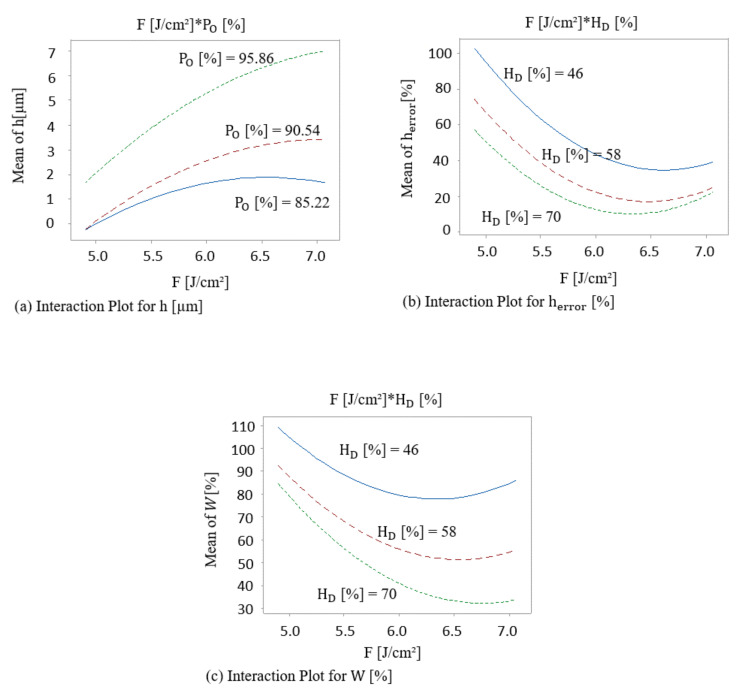
Interaction plots of F (J/cm^2^)·$P_{O}$ (%) for the (**a**) structures height, and of F (J/cm^2^)·$H_{D}$ (%) for (**b**) structure height error, and (**c**) surface waviness.

After checking the statistical significance of the model, important interactions of the factors that mostly affect the response were analyzed by using a graphical tool called interaction plots. These interaction plots are shown in Figure 7 and they are plotted from fitted values of predicted responses from the region of interest that consist of central and factorial points, excluding axial ones due to higher magnitude of prediction error. Figure 7a shows that the change in the mean response (height), from a low to a high level of laser fluence factor, depend on the level of the other factor which is pulse overlap. Additionally, the effect of laser fluence on the mean height of the structure is stronger for higher values of pulse overlap (Po), which is visible by the increasing slope of the curves with increasing Po level. The maximum height of the structures is reached when both fluence and pulse overlap are kept at a relatively high-level of 7.07 J/cm^2^ and 95.86%, respectively. Moreover, Figure 7b,c shows the presence of interaction between the fluence and the hatch distance, which significantly affect the structure height error as well as surface waviness. It is worth mentioning that an increase of both the fluence and the hatch distance tends to decrease the structures height error as well as the surface waviness and thus to improve homogeneity. However, the increase of fluence is effective only until the negative vertex displayed in Figure 7b,c. After this critical point, the curve changes its slope, which means that a further increase of the laser fluence may have an opposite effect in the structure height and will decrease homogeneity. Indeed, a very high value of fluence can negatively affect the quality and the height of the fabricated structures, as the accumulated energy may increase, and the possibility of uncontrolled overmelt occurs, which was already shown in previous investigations [50].

**Figure 8:**
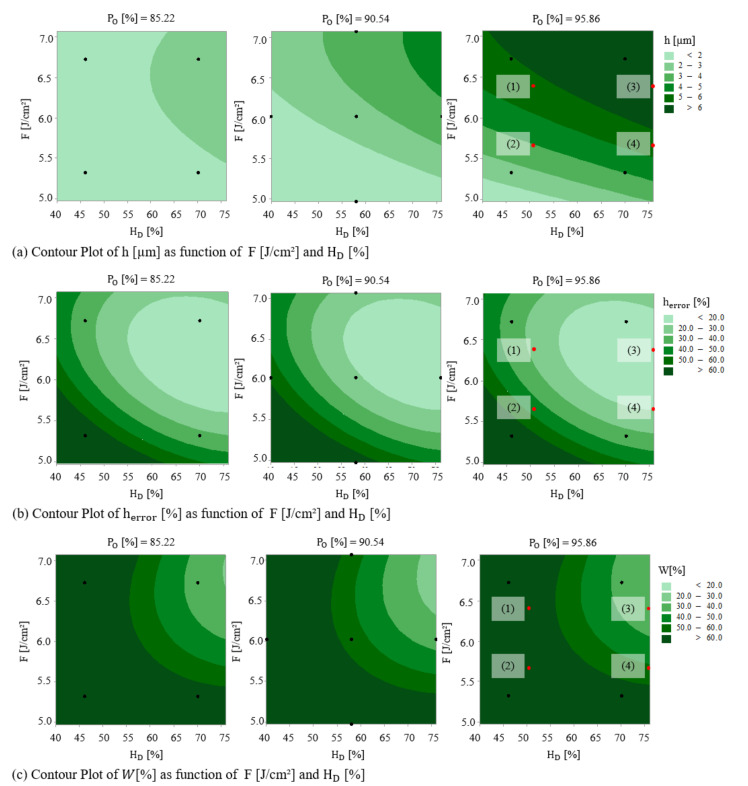
Contour plots of (**a**) height, (**b**) height error, and (**c**) surface waviness.

Finally, all the effects of the individual main effect of each factor, their quadratic terms, as well as their interactions can be summarized with the help of 2D contour plots, which are generally the graphical representation of the regression equation. Therefore, the accuracy of the contour plot depends on how well the model represents the true relationships between the variables. Each response surface presents the effect of laser fluence and hatch distance on structure height, height error, and surface waviness, while pulse overlap is held at a fixed specific level of 85.22%, 90.54%, and 95.86%. Each contour plot in Figure 8 has the dominant characteristic of the non-linear surface (fan-shaped, in this case). Such non-linearity implies a strong *X*_1_ × *X*_3_ (fluence with hatch distance) interaction effect, whereas it can be concluded from Figure 8b,c that the pulse overlap does not have a significant effect on the interaction between laser fluence and hatch distance, which indicates that interactions *X*_2_ × *X*_3_ and *X*_1_ × *X*_2_ are negligible. Moreover, the response surface plots exhibit a saddle shape, which means that any increase or decrease of fluence from the saddle peak results in a decrease of each of the measured response factors. For instance, in the case of Po = 85.22%, H_D_ = 60%, and for fluences from 6.5 J/cm^2^ and higher, the structure height saturates and even slightly reduces, because of material overmelting, as previously reported in [50].

Similarly, for Po = 90.54%, H_D_ = 55%, and for fluences from 6.5 J/cm^2^ and higher, the structure height error reaches its minimum and then again increases. The same behavior is observed for surface waviness by increasing laser fluence more than 6.5 J/cm^2^ at Po = 90.54% and H_D_ = 72%. It is attributed to the negative effect of the quadratic term (*X*_1_ × *X*_1_) of the fluence. On the other hand, the quadratic term (*X*_3_ × *X*_3_) of the hatch distance has a positive effect on the response. The best values of response for each of the structure height (>6 µm), height error (<20%), and surface waviness (~24%) are in the upper right corner of the plot, which corresponds with high values of both laser fluence (*X*_1_) and hatch distance (*X*_3_). The lowest values of the structures height (<2 µm), height error (>60%), and surface waviness (>60%) are in the lower-left corner of the plot, which corresponds to low values of both *X*_1_ and *X*_3_.

The tendency that the increase of levels of varied factors of DLIP process improves the response in the form of patterned surface homogeneity can be explained by redistribution of cumulative laser intensity that controls the quantity of molten and ablated material during the movement of the substrate in the x and y directions. Nevertheless, the factors involved in the DLIP process have an optimal level after which a further increase will lead to worse response values, which means that the homogeneity will be damaged and the height of the structure will collapse. This is in agreement with a generally accepted theory for near-surface melt dynamics during laser processes, where the melt flow (based on Marangoni convection) is considered as the main driving force in the microstructure formation besides recoil and plasma pressure [22,24]. In this case, the excess of deposited intensity on the processed surface that leads to uncontrolled melt of the material that is further explained in [50].

**Figure 9:**
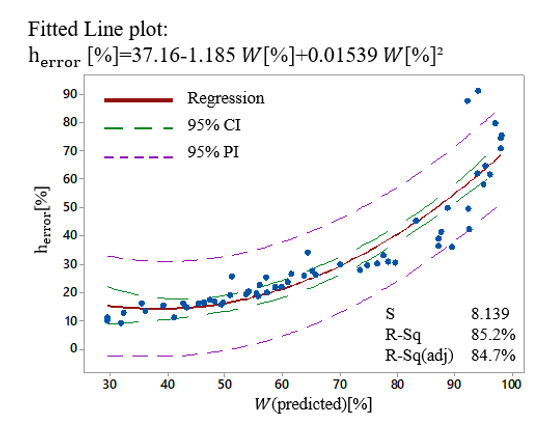
Correlation fitted line plots between height error (*h_error_* (%)) and surface waviness (*W* (%)).

Since both error contour plots (Figure 8b,c) showed that the low error zones can be found for high laser-fluence and hatch distance (upper right corner), a correlation graph between the structure height error and surface waviness was realized, permitting to estimate the strength of this relationship. The correlation graph presented in Figure 9 shows that a high correlation exists between the structure height error and surface waviness. The fitted equation for the quadratic model that describes the relationship between *h_error_* (%) and *W* (%) is:

(15) $$W\left(\%\right)=\;37.16\;-\;1.185\;h_{error}\left(\%\right)+\;0.01539\;h_{error}{\left(\%\right)}^{2}$$

The *R*^2^ in each of the correlation graphs shows that the developed model can explain >80% variability in the response. However, this statistically significant relationship does not imply that the height error (*h_error_*) causes surface waviness (*W* (%)). Nevertheless, since the model fits the data well, this equation can be used to predict *h_error_* (%) for a value of W (%), or find the settings for *W* (%) that correspond to a desired value or range of values for *h_error_* (%).

### 3.2. Model Validation

**Figure 10:**
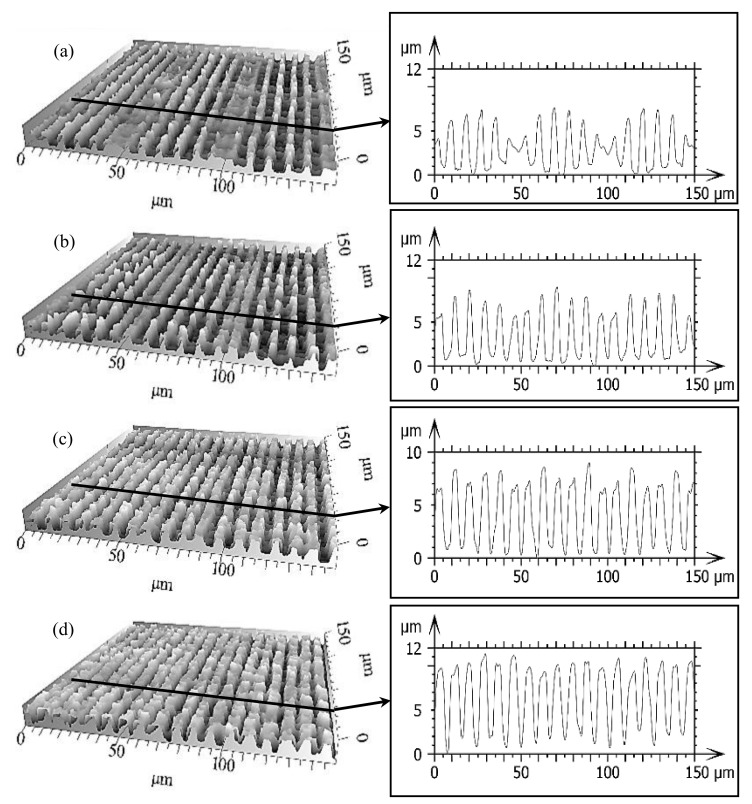
Confocal microscopy pictures of the nanosecond DLIP structures produced on 100Cr6 steel using spatial period Λ = 8.50μm, and the corresponding processing parameters: F = 5.69 J/cm^2^, P_O_ = 95.86%, H_D_ = 52% (**a**), F = 6.4 J/cm^2^, P_O_ = 95.86%, H_D_ = 52% (**b**), F = 5.69 J/cm^2^, P_O_ = 95.86%, H_D_ = 76% (**c**), and F = 6.4 J/cm^2^, P_O_ = 95.86%, H_D_ = 76% (**d**). The insets show the cross-section profiles of the corresponding topographies.

To confirm the validity and accuracy of the developed model, additional experiments were done in triplicates according to manually chosen parameters (marked by red dots in Figure 8). The DLIP structuring runs were conducted in the same conditions as in the previous described experiment. The corresponding topographies with extracted profiles are presented in Figure 10.

**Figure 11:**
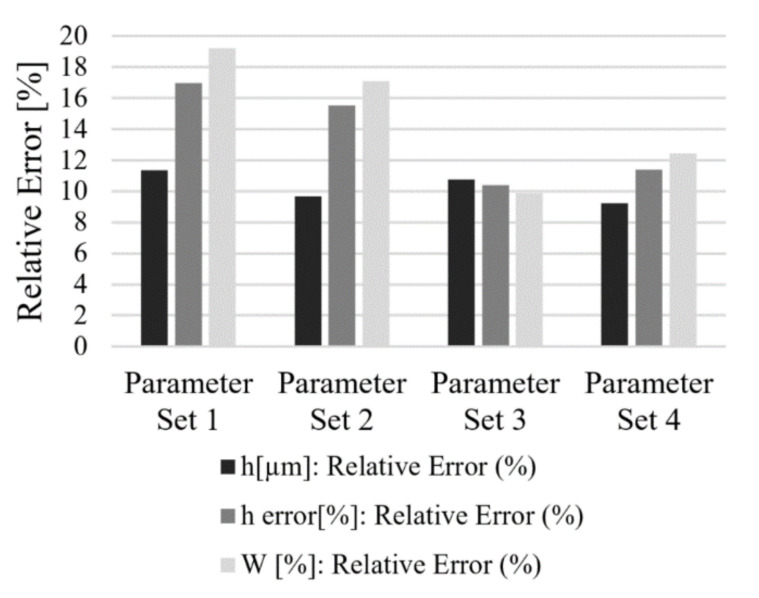
Bar charts summarizing relative error between model-predicted and verification experiments with parameter sets: (**1**) F = 5.69 J/cm^2^, P_O_ = 95.86%, H_D_ = 52%, (**2**) F = 6.4 J/cm^2^, P_O_ = 95.86%, H_D_ = 52%, (**3**) F = 5.69 J/cm^2^, P_O_ = 95.86%, H_D_ = 76%, and (**4**) F = 6.4 J/cm^2^, P_O_ = 95.86%, H_D_ = 76%.

After the analysis, the relative error between the calculated surface quality parameters and the experimental values was calculated for the structures height, height error, and surface waviness (Figure 11). The results show that the predicted value of the structure height was very close to the experimental results with a relative error varying between 8.5% and 11% for different parameter sets. Additionally, the relative error for surface structures error varied between 10% and 20%. In consequence, the results indicate that the prediction model achieved in the present study is reliable.

### 3.3. Multi-Objective Optimization

**Figure 12:**
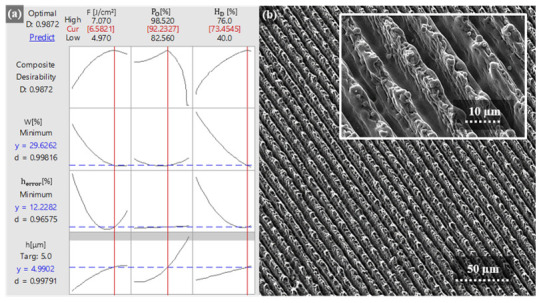
Summary of the multi-objective optimization process where in (**a**) Minitab Response optimizer is presented and in (**b**) the Scanning electron micrograph visualizes line-like DLIP microstructures produced on 100Cr6 steel using the processing parameters predicted by the Response optimizer.

Finally, a multi-objective optimization was performed employing the response optimizer of Minitab, in order to identify the optimum process parameters that minimize the error in structures height and waviness. The optimization plot shows the effect of each process parameter (the model factors; see columns in Figure 12a) on the surface texture characteristics (the responses or composite desirability; see rows in Figure 12a). The vertical red lines on the graph represent the current factor settings, and the numbers displayed in red at the top of a column show those current factor level settings. In the same manner, the horizontal blue lines and the corresponding blue numbers represent the response values for the current factor level. Moreover, the composite desirability value denoted by D shows how the response from the predicted factor levels met the initial requirements. Furthermore, the optimization plot also allows us interactively change and adjust the input variable settings to perform sensitivity analysis and to search for more desirable or improved solutions.

The predictive nature of the optimization plot is tested against an experimental example, where the aim was to structure the surface with minimal waviness and line-like microstructures with 5 µm in height which should have minimal height error. In order to reach a desired surface quality, the developed model suggests to use the process parameters of F = 6.58 J/cm^2^, P_O_ = 92.23%, and H_D_ = 73.45% with the composite desirability value equal to 98.7%. Afterwards, the predicted and optimized parameters were used in the structuring process. The resulting surface topography is visualized in the scanning electron micrograph presented in Figure 12b. This surface topography is characterized by a 5.43 µm structure height, ~13.78% height error, and ~25.83% waviness, thus, similar well enough to the predicted values of 4.99 µm, 12.23%, and 29.63%, respectively. Consequently, this demonstrates that the developed model is statistically reliable and can be used for prediction and optimization of the processing parameters.

## 4. Conclusions

The present work deals with the experimental investigation and analysis of DLIP processes on bearing steel material (100Cr6) using a laser texturing strategy by a pulsed nanosecond infrared laser. The main goal was to investigate the fundamental relationship between the process parameters and resulting surface texture quality measures by means of a central composite design (CCD) method. This includes the development of an empirical model which is expressed by second-order polynomial equations providing linear and quadratic interaction effects of laser processing parameters influencing measured topographical properties. The developed model was able to accurately describe (with an accuracy of more than 80%) the correlation of varied factors and the measured response. The data analysis using the Pareto charts showed that both laser fluence and pulse overlap process parameters have the highest influence on the resulting structure depth. Furthermore, it was found that the laser fluence and hatch distance affect predominantly the structure height error and surface waviness of the fabricated structure. Moreover, the model allowed to identify cross-correlation between laser fluence and pulse overlap in the case of the structures’ height response as well as between laser fluence and hatch distance in each of surface waviness and structure height error. This implies that the change in the mean response from low to high level of a factor depend on the level of the other factor. Furthermore, the model allows to predict optimal process parameters for fabricating target surface textures with specific quality features. Finally, the model helped to understand how the certain undesired topographical values, such as structure height error and waviness, can be reduced in order to improve the homogeneity of the periodic surface structures.

## Figures and Tables

**Table 1:** Factors and their adopted (uncoded) values at different coded levels.

$\mathrm{Factors}\;\left(Y\right)$	Symbol	Coded:	−1.5	−1	0	+1	+1.5
		Uncoded Values of Coded Levels:					
Peak-Fluence: F (J/cm^2^)	*X*_1_		4.97	5.32	6.02	6.72	7.07
Pulse Overlap: P_O_ (%)	*X*_2_		82.56	85.22	90.54	95.86	98.52
Hatch Distance: H_D_ (%)	*X*_3_		40	46	58	70	76

**Table 2:** Contribution of significant main factors (in %), their interactions and quadratic effects of factors from the model for each of the responses, with *X*_1_ = Fluence, *X*_2_ = Pulse overlap and *X*_3_ = Hatch Distance.

Response	*X*_1_	*X*_2_	*X*_3_	*X*_1_ *X*_1_	*X*_2_ *X*_2_	*X*_3_ *X*_3_	*X*_1_ *X*_2_	*X*_1_ *X*_3_	*X*_2_ *X*_3_	Error
Factors										
*h* (µm)	17.5	46.56	6.96	1.74	9.49	0.13	2.69	0.25	0.02	14.78
$h_{\mathrm{error}}$ (%)	31.64	0.05	34.24	15.25	0.06	3.99	0.37	2.02	0.01	12.02
*W* (%)	17.75	0.03	53.5	6.43	2.15	2.2	0.15	1.8	0.96	14.67
